# The NALP3/Cryopyrin-Inflammasome Complex is Expressed in LPS-Induced Ocular Inflammation

**DOI:** 10.1155/2008/614345

**Published:** 2008-08-25

**Authors:** José F. González-Benítez, Marco A. Juárez-Verdayes, Sandra Rodríguez-Martínez, Mario E. Cancino-Diaz, Francisco García-Vázquez, Juan C. Cancino-Diaz

**Affiliations:** ^1^Microbiology Department, Escuela Nacional de Ciencias Biológicas, Instituto Politécnico Nacional, Mexico City 11340, Mexico; ^2^Immunology Department, Escuela Nacional de Ciencias Biológicas, Instituto Politécnico Nacional, Mexico City 11340, Mexico; ^3^Pathology Department, Instituto Nacional de Pediatría, Mexico City 14410, Mexico

## Abstract

In the inflammosome complex, NALP3 or NALP1 binds to ASC and activates caspase-1 which induces IL-1*β*. In murine LPS-induced ocular inflammation, the production of IL-1*β* is increased. We suggest that NALP3- or NALP1-inflammasome complex can be participating in the LPS-induced ocular inflammation. In this work, eye, brain, testis, heart, spleen, and lung were obtained from C3H/HeN mice treated with LPS for 3 to 48 hours, and the expression of NALP1b, NALP3, ASC, caspase-1, IL-1*β*, and IL-18 was determined. Infiltrated leukocytes producing IL-1*β* in the anterior chamber were found at 12-hour posttreatment. A high upregulated expression of NALP3, ASC, caspase-1, IL-1*β*, and IL-18 was found at the same time when infiltrated leukocytes were observed. NALP1b was not detected in the eye of treated mice. NALP3 was also overexpressed in heart and lung. These results suggest that NALP3-, but not NALP1-inflammosome complex, is participating in the murine LPS-induced ocular inflammation.

## 1. INTRODUCTION

The innate immunity is the first barrier in the protection against ocular infections. The Toll-like receptors (TLRs), that belong to pattern recognition receptors (PRRs) family, have been the molecules involved in the recognition of pathogen-associated molecular patterns (PAMPs) that lead to ocular inflammatory process. Nevertheless, other PRRs such as Nod-like receptor (NLR) could also be participating in this phenomenon. The human genome database reveals that NLR family consist of at least 22 genes. Alternative names for this family include CATERPILLER, NOD, and NOD-LRR. The NLRs have three structural domains, LRR domain (carboxy-terminal), an intermediary NACHT (NBS; NOD) domain, and an effector domain (amino-terminal), which can be a pyrin domain (PYD), a caspase recruitment domain (CARD), or a baculovirus inhibitor of apoptosis protein repeat (BIR) domain [1].

Based on the phylogenetic history of NACHT domain (which is only present in NLR), and on the particular type of effector domain, the 22 NLR family members can be classified in two large subfamilies: the NODs (NLR family, CARD domain containing) also called NLRCs, and the NALPs (NLR family, pyrin domain containing) also called NLRPs. Through genomic database searches, 14 NALPs members have been identified in the human genome [1, 2]. As NALPs have been recently discovered, the participation of these molecules in the ocular inflammation remains unknown.

One well-established feature of NALPs is their ability to interact with apoptosis-associated speck-like protein (ASC) through a PYD-PYD interaction. ASC encodes a 22-kDa protein that contains a carboxy-terminal CARD and an amino-terminal PYD, suiting as an adaptor molecule between PYD- and CARD-containing proteins. An interaction between ASC and NALPs was shown initially for NALP1 (Nlrp1/DEFCAP/NAC/CARD7) and subsequently also found for NALP2 and NALP3 (Nlrp3/PYPAF1/Cryopyrin/CIAS1). The PYD of ASC interacts with the PYD of several NALPs, whereas ASC's CARD recruits the CARD of procaspase-1 for its activation [3, 4]. The active caspase-1 was found to be required for the production of active IL-1*β* and IL-18 in activated macrophages and monocytes [5]. When NALP and ASC interact with activate caspase-1 or caspase-5, they create an intracellular complex termed inflammosome [1, 2].

More than one type of inflammasomes have been identified up to date. NALP1-inflammasome is composed of NALP1, ASC, caspase-1, and caspase-5, whereas the NALP2/3-inflammasomes contain NALP2 or NALP3, ASC and caspase-1. A fourth inflammasome is composed of IPAF, ASC, and caspase-1 [1, 2]. NALP12 does not form inflammasome complex but it participate in inflammation as a negative regulator interfering with IRAK-1 function, resulting in the repression of TLR signalling [6].

LPS-induced ocular inflammation is a model to study molecular mechanisms involved in murine uveitis, where the expression of proinflammatory cytokines such as IL-1*β*, TNF*α*, and IL-6 is present [7]. We suggest that an inflammasome complex could be participating in the murine LPS-induced ocular inflammation. In this work, mRNA expression of NALP1b, NALP3, NALP12, ASC, caspase-1, IL-6, TNF*α*, IL-18, and IL-1*β* in the eye of mice treated with LPS was studied.

## 2. MATERIALS AND METHODS

### 2.1. Mouse strain

C3H/HeN mice (Harlan, Mexico City, Mexico) susceptible to ocular inflammation by endotoxin were used. All mice were healthy, 6–8 weeks old, and did not present any ophthalmologic alteration. All mice were maintained in aseptic conditions during the treatment.

The experimental protocols were performed in accordance to the Instituto Politécnico Nacional statements for the use of animals in research.

### 2.2. LPS-induced ocular inflammation

To generate LPS-induced ocular inflammation, C3H/HeN mice received a footpad injection of 200 *μ*g of *Escherichia coli* K-235 LPS (Sigma Chemical Company, Mo, USA) in 50 *μ*L of phosphate-buffered saline solution (PBS). The mice were killed by cervical dislocation at 3, 6, 12, 24, and 48 hours posttreatment and the eye, brain, testis, heart, spleen, and lung were obtained from each mouse. As control, five mice received a footpad injection of 50 *μ*L sterile PBS and killed after 48 hours. Groups of 5 treated mice were included in each time of treatment. From each mouse, one eye was fixed in formaldehyde for histology analysis and the other one was treated with Trizol (Invitro, Carlsbad, Calif, USA) reagent for RT-PCR analysis.

### 2.3. Histological analysis

To verify if infiltration of leukocytes in the eyes of treated mice occurred, fixation of the whole eye in buffered formaldehyde (3.5 g NaH_2_PO_4_ anhydrous, 6.5 g NaH_2_PO_4_, and 40% formaldehyde) was carried out and embedded in paraffin. Sections of 3 *μ*m slides were stained with haematoxylin-eosin. For quantification of infiltrated cells, the observed cells per field of view (40X) were counted, and the mean value was reported.

### 2.4. Immunohistochemical analysis

The immunohistochemical analysis was developed in 3.0 *μ*m sections of ocular and testis tissues fixed with buffered formaldehyde. Rabbit polyclonal IgG antimouse IL-1*β* (Santa Cruz Biotechnology, Santa Cruz, Calif, USA) and irrelevant rabbit IgG (Santa Cruz Biotechnology) were used to assess expression of the proteins, followed by a secondary biotinylated antibody and streptavidin/peroxidase complex (LSAB+ Kit peroxidase, Dako Cytomation, Carpinteria, Calif, USA), and visualized with TACS-Blue Label (R&D System, Minn, USA) as chromogen and counterstained with Nuclear Fast Red counterstain (Sigma-Aldrich, Mo, USA).

### 2.5. RNA isolation and RT-PCR analysis

All studied organs were thoroughly washed in D-PBS to eliminate blood. Total RNA extraction was performed with TRIzol reagent and treated with DNAse I free of RNAse. For reverse transcriptase (RT) reaction, total RNA (3 *μ*g) and 0.5 *μ*g of oligo-(dT)_15-18_ (Invitrogen) were denatured at 70°C for 10 minutes, and 1X single strand buffer, 0.5 mM DTT, 500 *μ*M of each dNTPs, and 200U of MMLV reverse transcriptase (Invitrogen, Ohio, USA) were added. The RT reactions were performed at 42°C for 1 hour. The PCR reactions were prepared with 1 *μ*L of the cDNA, 1X buffer, 1 mM MgCl_2_, 200 *μ*M of each dNTPs, 1U of TaqDNA polymerase, and 0.2 *μ*M of each NALPs, ASC, caspase-1, IL-1*β*, IL-18, and *β*-actin primers (see Table 1). Optimal PCR conditions were 30 cycles of 92°C 30 seconds, 60°C 30 seconds, and 72°C 30 seconds.

To semiquantitate PCR products, the intensity of the amplified bands was analyzed with the AlphaImager software. Band intensities were normalized with the corresponding *β*-actin signal, and relations NALPs/*β*-actin, ASC/*β*-actin, caspase-1/*β*-actin, and interleukines/*β*-actin were calculated. The results were analyzed by Kruskal-Wallis and Turkey statistical tests.

## 3. RESULTS

### 3.1. Analysis of ocular inflammation in mice treated with LPS

**Figure 1:**
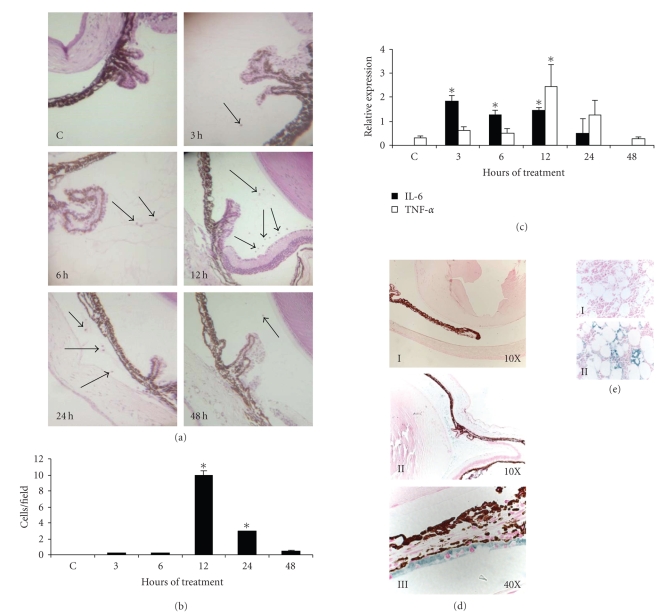
*Histological and molecular analysis of the LPS-induced ocular inflammation*. C3H/HeN mice were inoculated in the footpad with 200 *μ*g/50 *μ*L of LPS and the inflammation was analyzed at 3, 6, 12, 24, and 48 hours. Control mice (C) received a footpad injection of 50 *μ*L of sterile PBS. (a) histological analysis of leukocyte infiltration in the anterior chamber stained with hematoxylin-eosin (40x). The arrows point infiltrated cells. The figure shows a representative result of five mice studied in each time. (b) average of infiltrated leukocytes observed in (a). (c) average of semiquantitative analysis of IL-6 and TNF*α* mRNA expression. Asterisk indicates significant difference compared with the control (*P* < .05) analyzed by Kruskal-Wallis and Turkey tests. (d) ocular expression of IL1*β* protein in treated and untreated mice. Immunostaing of IL-1*β* of untreated mouse (I) and treated mice at 12 hours posttreatment (II and III). (e) testis tissue was used as positive control of IL1*β* expression; irrelevant antibody (I), IL-1*β* antibody (II). Blue staining means positive signal.

Histological and molecular analyses were done to evaluate the ocular inflammation of mice treated with LPS, therefore leukocytes infiltrated in the anterior chamber and proinflammatory cytokines expression were determinated. In the eyes of LPS-treated mice, there were leukocytes infiltrated in the anterior chamber from 3 to 48 hours of treatment (see Figure 1(a)) and at 12 hours the mean value of infiltrated leukocytes was the highest (ten cells per field; *P* < .05; see Figure 1(b)).

To confirm ocular inflammation in the eye of treated mice, the mRNA expression of the proinflammatory cytokines IL-6 and TNF*α* was determined. IL-6 mRNA was highly expressed in LPS-treated mice and semiquantitative analysis showed a significant increase of IL-6 at 3, 6, and 12 hours posttreatment (see Figure 1(c)). In treated mice, the highest expression of TNF*α* mRNA was observed at 12 hours (see Figure 1(c)). At this time, it was also observed that the infiltrated leukocytes were producing and secreting IL-1*β* (see Figure 1(d)). These results demonstrate that LPS induced ocular inflammation.

### 3.2. Expression analysis of NALP1b and NALP3 in mice treated with LPS

**Figure 2:**
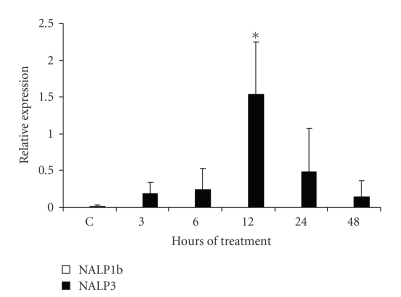
*Semiquantitative analysis of NALP3 and NALP 1b mRNA expression in the eye of LPS-treated mice*. Mice were treated with LPS during 3, 6, 12, 24, and 48 hours. NALP3 and NALP1b mRNA expressions were determined by RT-PCR. Average of 5 mice analyzed in each assayed time is reported. Asterisk indicates significant difference compared with the control (*P* < .05) analyzed by Kruskal-Wallis and Turkey tests.

To analyse the type of inflammasome complex present in the eye of treated mice, expression of NALP1b and NALP3 was determined. NALP1b mRNA was not found, neither in the eye of the treated mice, nor in controls (see Figure 2). In contrast, NALP3 expression was detected in the eye of treated mice, and its highest expression was observed at 12 hours posttreatment (*P* < .05; see Figure 2). These results demonstrate that there is a differential expression of NALP1b and NALP3 in the LPS-induced ocular inflammation.

**Figure 3:**
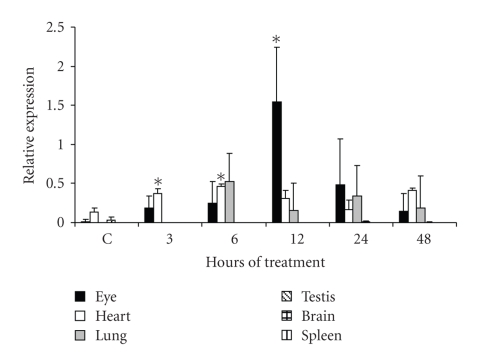
*Semiquantitative analysis of NALP3 mRNA expression in the eye, heart, lung, testis, brain, and spleen of LPS-treated mice*. NALP3 mRNA expression was determined by RT-PCR in the organs of mice treated with LPS during 3, 6, 12, 24, and 48 hours. Average of 5 mice analyzed in each assayed time is reported. Asterisk indicates significant difference compared with control (*P* < .05) analyzed by Kruskal-Wallis and Turkey tests.

In the other studied organs, NALP3 mRNA was detected in heart (from 3 hours posttreatment) and lung (from 6 hours posttreatment) but not in testis, brain, and spleen (see Figure 3). Although the highest NALP3 expression occurred in the eye at 12 hours posttreatment, NALP3 arose earlier in heart and lung (3 and 6 hours, resp.).

### 3.3. Expression analysis of ASC, caspase-1, IL-1*β*, and IL-18 in the eye of LPS-treated mice

**Figure 4:**
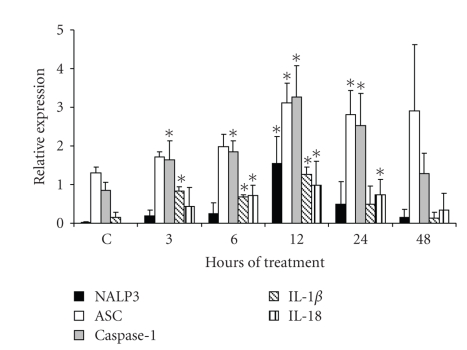
*Expression analysis of NALP3-inflammasome components in the eye of LPS-treated mice*. Semiquantitative RT-PCRs were performed for the analysis of NALP3, ASC, caspase-1, IL-1*β*, and IL-18 expression in the eye of LPS-treated mice. Average of 5 mice analyzed in each assayed time is reported. Asterisk indicates significant difference compared with the control (*P* < .05) analyzed by Kruskal-Wallis and Turkey tests.

As NALP3 expression occurred in the eye, and as an increase of its expression was induced by LPS, the expression of the other inflammasome components (ASC, caspase-1, IL-1*β*, and IL-18) in this organ was determined. The ASC mRNA expression was observed in both nontreated and treated mice but the highest expression was found at 12 hours posttreatment (see Figure 4). The expression of caspase-1 was gradually increased from 3 hours, and at 12 hours it showed its highest expression (see Figure 4). The expression of IL-1*β* was upregulated from 3 hours posttreatment, and its highest expression happened at 12 hours posttreatment as occurred with the protein (see Figures 4 and 1(d)). Similar results were observed for the IL-18 expression (see Figure 4).

### 3.4. Expression of NALP12 (Monarch 1) and NALP5 in the eye LPS-treated mice

**Figure 5:**
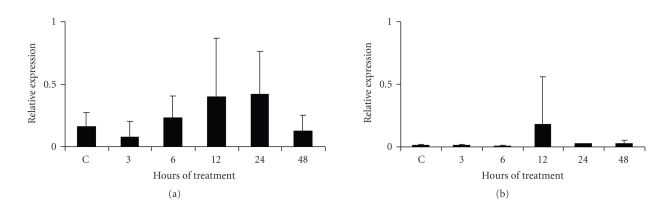
*Semiquantitative analysis of NALP12 and NALP5 mRNA expression in the eye LPS-treated mice*. NALP12 (panel (a)) and NALP5 (panel (b)) mRNA expression were determined by RT-PCR. Mice were treated with LPS during 3, 6, 12, 24, and 48 hours. Average of 5 mice analyzed in each assayed time is reported. Analysis of Kruskal-Wallis test showed no statistical significance.

As NALP12 (Monarch 1) is a molecule that can negatively regulate the inflammasome complex, the participation of this molecule in the LPS-induced ocular inflammation was investigated. A slight induction in the expression of NALP12 due to LPS-treatment was observed at 12 and 24 hours, but without statistical relevance (*P* > .05; see Figure 5(a)).

To assess if the LPS treatment can also modify the expression of other NALPs that are not involved in the inflammasome complex, such as NALP5, the expression of this molecule was determinated. The eye of untreated mice did not express NALP5, and the LPS induced slight expression of it at 12 hours posttreatment, but again without statistical relevance (*P* > .05; see Figure 5(b)).

## 4. DISCUSSION

In this work, we observed that LPS induced the mRNA expression of IL-1*β*, IL-6, TNF-*α*, and IL-18 as well as the secretion of IL-1*β* by the leukocyte infiltration in the anterior chamber of the eye, confirming an ocular inflammation process. Therefore, we searched if the expression of NALP3, NALP1b, ASC, and caspase-1 correlates with the expression of IL-1*β* and IL-18 in the LPS-induced ocular inflammation model.

NALP3 forms a multiprotein complex called “inflammasome,” which contains ASC and caspase-1, and this complex promotes caspase-1 activation and consequently the processing of pro-IL-1*β* and IL-18 in macrophages cultures [2]. We found that the NALP3 mRNA expression was increased in the eye of mice treated with LPS at 12 hours. The expression of NALP1b was found neither in treated nor in control mice, suggesting that the NALP3-inflammosome complex, but not the NALP1b-inflammosome complex, could be associated with the LPS-induced ocular inflammation. The NALP3 expression has been documented predominantly in monocytes, granulocytes, and chondrocytes [8]. The murine NALP3 mRNA expression was found initially in peripheral blood leukocytes, consistent with the postulation of an inflammatory function. NALP3 has also been detected in the eye and skin of healthy mouse [9]. Nevertheless, this is the first report that shows the increase of the NALP3 expression in the LPS-induced ocular inflammation. It is important to highlight that the overexpression of NALP3 correlated with the time leukocytes was found infiltrated producing IL-1*β*. In vitro studies have demonstrated that primary human monocytes stimulated with LTA, LPS, or poly(I:C) elicited a robust induction of NALP3 expression [10]. With our results, we show that LPS can induce in vivo the expression of NALP3 in the eye.

This is also the first report that shows that LPS induces the expression of NALP3 in heart and lung but not in spleen, nevertheless it has been reported that NALP3 is not expressed in lymphoid and skin tissues of healthy mouse [11] but that NALP1 does. Chu et al. reported that NALP1 mRNA is expressed in healthy organs, such as heart, thymus, spleen, kidney, liver, lung, and peripheral blood lymphocytes [12]; we are reporting that NALP1 is not expressed in the eye of healthy mice, but even after treatment with LPS it remains absent.

We found that the expression of NALP3 in the eye is delayed respect heart's and lung's. About it, we suggest that the hemato-ocular barrier that could delay the LPS arrival to the ocular tissues interfering in the time NALP3 is induced. The early expression of NALP3 in heart and lung could contribute to the effect of septic shock by LPS.

NALP3-deficient mice have been generated to investigate the role of NALP3 in inflammatory responses to pathogen-derived molecules. NALP3-deficient macrophages do not produce active IL-1*β* in response to LPS, Pam3 CSK4 (TLR2 agonists), *Staphylococcus aureus, Listeria monocytogenes* [13], bacterial RNA, and the imidazoquinoline compounds R837 and R848 (TLR7 and TLR8 agonists) [14]. Nevertheless, NALP3-deficient macrophages, but not the IPAF-deficient macrophages, can produce IL-1*β* when stimulated with *Salmonella*. On the other hand, ASC-deficient macrophages do not produce IL-1*β* with any of the TLR agonists and cited bacteria. These results indicate that the adaptor ASC is the principal molecule for both NALP- and IPAF-inflammasome complexes [4, 15]. ASC is abundantly expressed in epithelial cells, leukocytes, hair follicles, and peripheral blood lymphocytes [16], and in neutrophils located in severe inflammatory sites [17]. In the eye, we found that ASC is expressed, and that the LPS can increase its expression.

Caspase-1 is another component of the inflammasome complex useful for the activation of IL-1*β*. Caspase-1-deficient mice carry a defect in the production of mature IL-1*β* after stimulation with LPS. Furthermore, these mice are resistant to endotoxic shock [18]. Bacterial RNA can also induce the activation of caspase-1 trough NALP3 [14]. We found that caspase-1 was increased in the eyes of the mice treated with LPS at the same time when NALP3, ASC, IL-1*β*, and IL-18 showed their highest expression (at 12 hours). These results suggest that the high expression of NALP3, caspase-1, ASC, IL-1*β*, and IL-18 could be participating in the murine LPS-induced ocular inflammation.

In this report, we also analyzed the NALP12 expression in LPS-induced ocular inflammation. Although a slight increase in the expression of NALP12 in the eyes of treated mice was detected, statistically it was not relevant. This suggests that this molecule does not participate in the regulation of the LPS-induced ocular inflammation as occurs in monocytes and granulocytes stimulated with TNF*α* and other TLRs ligands [6].

NALP5 has not been involved in the inflammasome complex, but it has been related to reproduction because it has been detected in oocytes [19, 20]. So far, there are no reports about the expression of NALP5 in the eye. Although we did not find NALP5 expressed in the eye of the control mice, we found it barely detectable in some eyes of treated mice.

In summary, these results suggest that LPS induced a significant expression of NALP3-inflammasomme complex in the endotoxin-induced ocular inflammation.

## Figures and Tables

**Table 1:** Sequence of oligonucleotides.

Gene	Sequence	Size amplified (pb)
*β*-Actin	Fw: 5′-TGGAATCCTGTGGCATCCATGAAAC-3′	324
	Rv: 5′-TAAAACGCAGCTCAGTAACAGTCCG-3′	

NALP3	Fw: 5′-CTGTGTGTGGGACTGAAGCAC-3′	544
	Rv: 5′-GCAGCCCTGCTGTTTCAGCAC-3′	

NALP1b	Fw: 5′-TGGGATGGTTCTAGAAACGCC-3′	602
	Rv: 5′-AGGGTCCACTGATGTCACTCG-3′	

NALP5	Fw: 5′-CCTGAGGAATCCAGAATGTGC-3′	238
	Rv: 5′-GCTTCCACAGGCCAATTATCC-3′	

NALP12	Fw: 5′-GTACCAACTCCAACCTGATCG-3′	512
	Rv: 5′-GAAGTAGAGGCCAGATCTTTGC-3′	

ASC	Fw: 5′-AGACATGGGCTTACAGGA-3′	257
	Rv: 5′-CTCCCTCATCTTGTCTTGG-3′	

Caspase-1	Fw: 5′-TGAAAGAGGTGAAAGAATT-3′	424
	Rv: 5′-TCTCCAAGACACATTATCT-3′	

IL-1*β*	Fw: 5′-TGGGCCTCAAAGGAAAGA-3′	157
	Rv: 5′-GGTGCTGATGTACCAGTT-3′	

IL-18	Fw: 5′-CTGTACAACCGCAGTAATACGG-3′	264
	Rv: 5′-ACTCCATCTTGTTGTGTCCTGG-3′	

IL-6	Fw: 5′-TCTCTGGGAAATCGTGGAAAT-3′	308
	Rv: 5′-TGTATCTCTCTGAAGGACTCTG-3′	

TNF*α*	Fw: 5′-CCTCCCTCTCATCAGTTCTATGG-3′	238
	Rv: 5′-TGTCCCTTGAAGAGAACCTGG-3′	
